# Genetically Predicted Milk Intake Increased Femoral Neck Bone Mineral Density in Women But Not in Men

**DOI:** 10.3389/fendo.2022.900109

**Published:** 2022-06-20

**Authors:** Song Chen, Changhua Zheng, Tianlai Chen, Jinchen Chen, Yuancheng Pan, Shunyou Chen

**Affiliations:** ^1^ Department of Orthopedics, Fuzhou Second Hospital, Fuzhou, China; ^2^ Fujian Provincial Clinical Medical Research Center for First Aid and Rehabilitation in Orthopaedic Trauma (2020Y2014), Fuzhou, China; ^3^ Fuzhou Trauma Medical Center, Fuzhou, China; ^4^ Department of Cardiology Nursing, Fujian Medical University Union Hospital, Fuzhou, China; ^5^ The Third Department of Clinical Medicine, Fujian Medical University, Fuzhou, China

**Keywords:** milk intake, bone mineral density, mendelian randomization study, cause effect, female

## Abstract

**Background:**

Cow milk contains more calcium, magnesium, potassium, zinc, and phosphorus minerals. For a long time, people have believed that increasing milk intake is beneficial to increasing bone density. Many confounding factors can affect milk consumption, and thus the association described to date may not be causal. We explored the causal relationship between genetically predicted milk consumption and Bone Mineral Density (BMD) of the femoral neck and lumbar spine based on 53,236 individuals from 27 studies of European ancestry using the Mendelian randomization (MR) study. 32,961 individuals of European and East Asian ancestry were used for sensitivity analysis.

**Methods:**

A genetic instrument used for evaluating milk consumption is rs4988235, a locus located at 13,910 base pairs upstream of the *LCT* gene. A Mendelian randomization (MR) analysis was conducted to study the effect of selected single nucleotide polymorphisms (SNPs) and BMD. The summary-level data for BMD of the femoral neck and lumbar spine were obtained from two GWAS meta-analyses [‘Data Release 2012’ and ‘Data Release 2015’ in the GEnetic Factors for OSteoporosis Consortium (GEFOS)].

**Results:**

we found that genetically predicted milk consumption was not associated with FN-BMD(OR 1.007; 95% CI 0.991–1.023; P = 0.385), LS-BMD(OR 1.003; 95% CI 0.983–1.024; P = 0.743) by performing a meta-analysis of several different cohort studies. High levels of genetically predicted milk intake were positively associated with increased FN-BMD in Women. The OR for each additional milk intake increasing allele was 1.032 (95%CI 1.005–1.059; P = 0.014). However, no causal relationship was found between milk consumption and FN-BMD in men (OR 0.996; 95% CI 0.964–1.029; P = 0.839). Genetically predicted milk consumption was not significantly associated with LS-BMD in women (OR 1.017; 95% CI 0.991–1.043; P = 0.198) and men (OR 1.011; 95% CI 0.978–1.045; P = 0.523).

**Conclusion:**

Our study found that women who consume more milk have a higher FN-BMD. When studying the effect of milk consumption on bone density in further studies, we need to pay more attention to women.

## Introduction

In bones, osteoporosis is a common metabolic skeletal disorder that has a causal relationship with aging and is characterized by poor bone strength. The microarchitecture of bone tissues deteriorate, and the risk for fractures increases (1). There is a growing prevalence of osteoporosis globally due to the rapidly aging population worldwide. The International Osteoporosis Foundation recently released statistics that report that, on average, 1 in 3 women over 50 years of age and 1 in 5 men will sustain osteoporotic fractures in their lifetimes. This disease significantly impacts on patients’ emotional, physical, and financial health. It can result in permanent disability, poor quality of life for elderly patients, and heavy financial burdens that patients must bear in attending to the high cost of treatment they must endure (2). Osteoporosis is primarily diagnosed by measurements of bone mineral density (BMD), which is the most common method of determining this condition, either by Dual Energy X-Ray Absorption (DXA) or bone densitometry (3). Research on twins and families has shown that cross-sectional BMD is highly heritable (50-85%) (4, 5). Many methods have been explored in preventing osteoporosis, and the dairy diet has received the most attention.

Compared with any other typical food in the adult diet, cow milk contains more calcium, magnesium, potassium, zinc, and phosphorus minerals (6). It is widely acknowledged that an enriched diet consisting of milk products would reduce the chances of osteoporosis. Research has suggested that taking dairy products equivalent to at least three or four glasses of milk a day will reduce at least 20% of the costs associated with osteoporosis (6). Even though several studies have suggested that dairy products and milk consumption can prevent fractures and osteoporosis (7–9). Many studies believe that milk intake has nothing to do with increasing bone density (10, 11). Some studies even think that drinking milk more than two times a day will increase fracture risk by 50% (12). There is no evidence to suggest that dairy products have any causal effect on preventing osteoporosis, and research is still ongoing regarding the issue (13, 14). Finnish study reports that high calcium intake in older and younger women is positively associated with non-weight-bearing radius but not with weight-bearing tibia (15). Another study also showed that the intake of calcium-rich foods such as milk was positively related to radial bone density, and it seemed that non-weight-bearing bone density benefited from high calcium intake, while weight-bearing bones like the femur and spine benefited from physical activity (16). As most studies on milk consumption and BMD have been observational or experimental, it is difficult to determine whether confounding factors or reverse causality, eliminated by MR, is responsible for the result. As no studies have studied the causal association between milk consumption and BMD, we decided to make an MR study to investigate the causation. MR is a genetic epidemiological method that uses genetic variants as instrumental variables. Reverse causation and potential confounding factors can be eliminated by MR (13). Single nucleotide polymorphism sites (SNPs) are assigned randomly at conception, avoiding reverse causation bias and residual confounding (14).

Lactase is encoded by the lactase gene (*LCT*), secreted by small intestinal cells, used to break down milk sugar. There is a single nucleotide polymorphism (SNP) upstream of the *LCT* gene that is related to lactase persistence (the presence of lactase in adulthood) and the increased consumption of milk by the European population during the 20th century (17, 18). We used *LCT* gene variation as an instrumental variable to represent milk consumption and assessed the potential causal relationship between milk consumption and bone density.

## Methods

### Data Resources

The femoral neck and lumbar spine are the two common osteoporotic fractures sites of postmenopausal women and men 50 years or older. The summary-level data for BMD were obtained from a GWAS meta-analysis, which included 53,236 individuals from 27 studies of European ancestry. We extracted data on BMD in the femoral neck (n=49988) and lumbar spine (n=44731) from this GWAS meta-analysis (**Supplementary Table 1**). Measurement of BMD was recommended utilizing dual-energy X-ray absorptiometry. GWAS summary statistics for BMD were downloaded from ‘Data Release 2015’(http://www.gefos.org/?q=content/data-release-2015) in the GEnetic Factors for OSteoporosis Consortium (GEFOS). It is the latest summary statistics of BMD in the femoral neck and lumbar spine. Besides, GEFOS is an extensive international collaboration comprising numerous research groups. This organization regularly publishes large samples of bone mineral density-related GWAS data.

In addition, we included another GWAS meta-analysis, ‘Data Release 2012’ (http://www.gefos.org/?q=content/data-release-2012), in the GEFOS for sensitivity analysis. This meta-analysis provides BMDs in the femoral neck (n=34,910) and lumbar spine (n=34,632) in different genders, which including 17 genome-wide association studies (**Supplementary Table 2**). For further analysis, we study the causal effect of milk intake on BMD in different genders.

### Genetic Instrument

**Figure 1:**
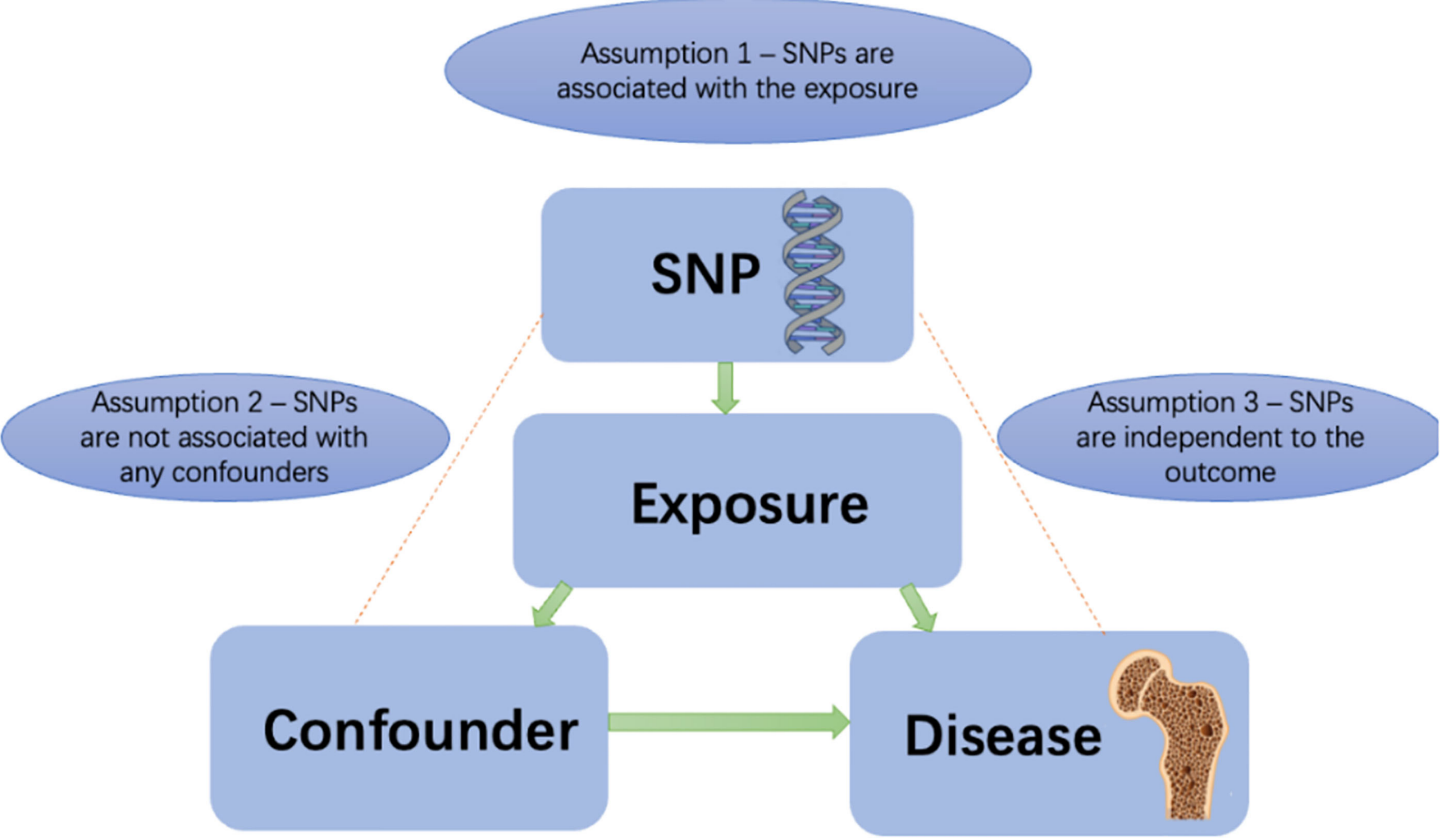
Principles of Mendelian randomization study.

Lactase breaks down the lactose in milk. People with lactase deficiency will experience diarrhea, bloating, and abdominal pain after eating cow’s milk (19). The milk intake of this group of people will be significantly lower than that of the regular group (20). A genetic instrument used for evaluating milk consumption is rs4988235, a locus located at 13,910 base pairs upstream of the *LCT* gene. Rs4988235(NC_000002.12:g.135851076G>A), located in the *MCM6* gene but with influence on the lactase *LCT* gene. Rs4988235 is one SNP associated with hypolactasia, more commonly known as lactose intolerance in European populations (21, 22). Several studies have demonstrated that rs4988235 is strongly associated with milk consumption among individuals in Europe, thus supporting the first MR assumption (17, 18) (**Figure 1**). Participants with the lactase persistent genotype TT/TC can digest more milk than do participants with the lactase nonpermanent genotype CC. During the EPIC-InterAct study, extra lactase persistence alleles (T) of rs4988235 increased daily milk consumption from 162 grams per day to 179 grams per day (11). In a Danish cohort of 73,715 individuals, weekly milk intake increased by 0.58 cups for each additional T allele of rs4988235 (18). According to genetic studies, the genetic variant rs4988235 is estimated to account for 2% of the variance in milk intake (18). This genetic variant has an F-statistic of 515, indicating an association between milk consumption and the variant (18). So using rs4988235 as an instrumental variable for milk intake is in line with the first assumption of MR studies (instrument variables are strongly correlated with exposure factors). For the second assumption of Mendelian randomization, we did not find any confounding factors associated with rs4988235 and affects BMD using phenoscanner database (http://www.phenoscanner.medschl.cam.ac.uk). The third assumption is that the instrumental variables affect outcomes only through causal pathways of exposure of interest and cannot be checked (23).

### Statistical Analysis

To calculate the ratio estimate for rs4988235, we divided the resultant beta coefficient by the beta coefficient for milk consumption. For each additional increase in milk intake, ORs and 95% CIs were computed for the T-allele of rs4988235, which increases milk intake. The meta-analysis used the MR random-effects model to pool individual outcomes. The analyses were performed using in RStudio version 1.4.1717. The following R packages were used during the study: TwoSampleMR package; meta package; forestplot package; ggplot2 package; grid package.

## Results

**Figure 2:**
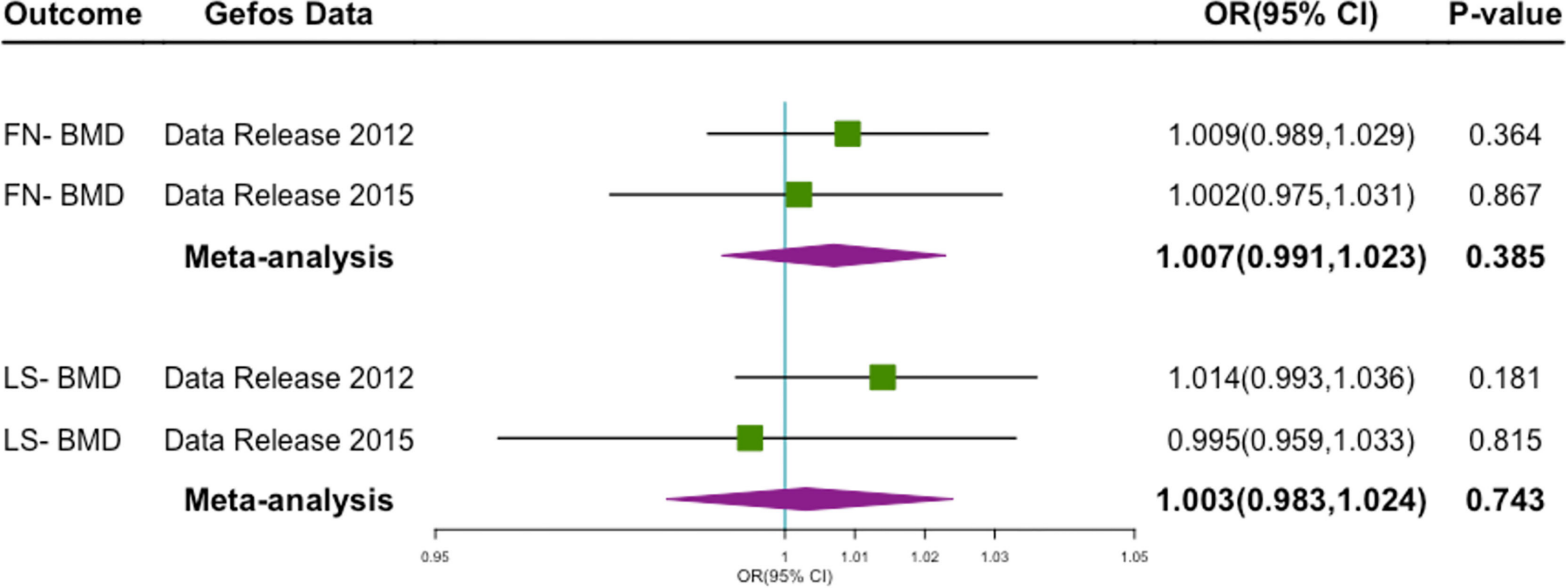
Forest plot of MR study using genetic instruments with FN-BMD, LS-MD, OR, odds ratio; CI, confidence interval; FN-BMD, femoral neck Bone Mineral Density; LS-MD, lumbar spine Bone Mineral Density.

By performing a meta-analysis of several different cohort studies, we found that genetically predicted milk consumption was not associated with FN-BMD(OR 1.007; 95% CI 0.991–1.023; P = 0.385), LS-BMD(OR 1.003; 95% CI 0.983–1.024; P = 0.743) (**Figure 2**).

**Figure 3:**
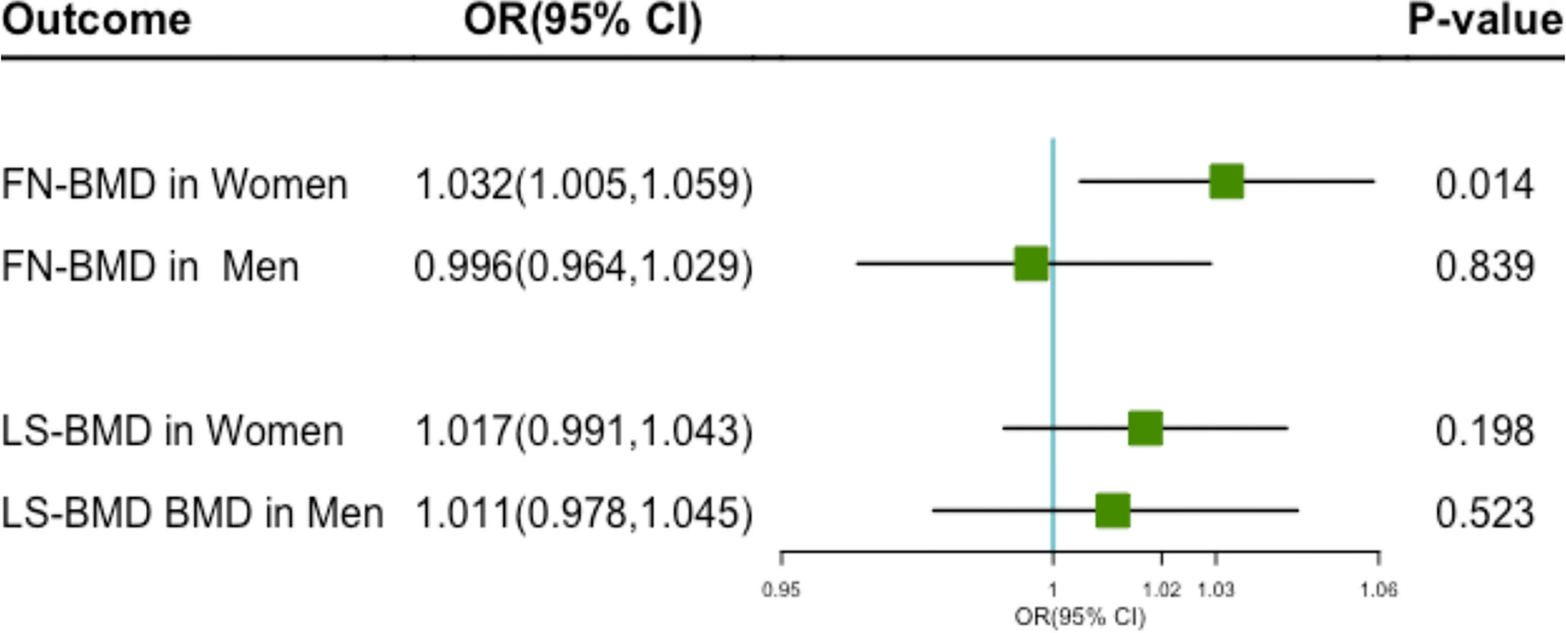
The plot of the MR study used genetic instruments with FN-BMD and LS-MD in different genders. OR, odds ratio; CI, confidence interval; FN-BMD, femoral neck Bone Mineral Density; LS-MD, lumbar spine Bone Mineral Density.

High levels of genetically predicted milk intake were positively associated with increased FN-BMD in Women (**Figure 3**). The OR for each additional milk intake increasing allele was 1.032 (95%CI 1.005–1.059; P = 0.014). However, no causal relationship was found between milk consumption and FN-BMD in men (OR 0.996; 95% CI 0.964–1.029; P = 0.839). Genetically predicted milk consumption was not significantly associated with LS-BMD in women(OR 1.017; 95% CI 0.991–1.043; P = 0.198) and men (OR 1.011; 95% CI 0.978–1.045; P = 0.523) (**Figure 3**).

## Discussion

We used the MR design to study the causal relationship between genetically predicted milk consumption and BMD of the femoral neck and lumbar spine. To analyze the difference in this causal relationship in different genders, we also used the other data in GEFOS to do a gender stratification analysis. As far as we know, this is the first MR study to evaluate the relationship between milk consumption and BMD.

Throughout the world, milk is a widely consumed beverage, and it provides essential macro and micronutrients that are essential for the health and well-being of millions of individuals (24). For a long time, people have believed that increasing milk intake is beneficial to increasing bone density (25, 26). In a prospective cohort study, researchers measured the BMD of the radius and tibia with ultrasound equipment. They found that intake of dairy products may reduce the incidence of radial osteoporosis in Korean postmenopausal women, but there is no significant effect in the tibia (15). The study also confirmed that milk might affect on bone density in other parts of the body (15). Consumption of high-calcium skim milk can effectively reduce bone loss in the hip in postmenopausal Chinese women in Malaysia, which is consistent with our study (27). A review study of older women using regular or fortified milk reports significant changes in bone biomarkers and some changes in bone density but no reduction in fracture risk (28). Using genetic MR analysis, this study found that women who consume more milk have a higher FN-BMD. However, this causality was not seen in male FN-BMD and LS-BMD.

Milk intake has a positively correlated causal effect on FN-BMD in women but not in men. There may be the following reasons: First, osteoporosis may be influenced by childhood or teenage years. Adequate milk intake during adolescence may reduce osteoporosis in adulthood. As a result of consuming a serving of milk per week (low intake) during childhood compared to consuming more than one serving per day (high intake), bone mineral content was 5.6% lower in women aged 20-49 (29). 18-year-old men and 20-year-old women can reach 90% of their peak bone mass (30). Both women and men continue to gain a relatively small amount of bone mass; however, men do so more rapidly than women do. Second, the risk of osteoporosis is related to estrogen levels. Women’s estrogen levels decline rapidly in the years after menopause, and the rate of bone loss at this time is much faster than at any other time in their lives. In a follow-up study of up to 15 years, researchers found a linear decline of 1.67% per year in femoral neck bone loss in women aged 45-68 (31). These differences in bone loss between men and women may explain the difference in the causal benefit of milk consumption. Some studies support our overhead view. Calcium from dairy products (fortified with calcium) can increase the bone mineral density of Caucasian women by 0.7 to1.8%, but not for men (32).

Our analysis has several strengths. We utilize the largest summary statistics data of BMD, which could overcome limitations of conventional epidemiological study designs, such as confounding and reverse causality. It is more time-efficient and less expensive than RCT. There were some limitations in this study. Firstly, our datasets included the European populations, which limited the applicability of results to non-European populations. Secondly, MR’s linear effect assumption could not further investigate nonlinear causality (33). Thirdly, the possibility cannot be ruled out that genetic instruments used to represent milk intake might affect BMD in ways other than milk intake, thus contradicting the second and third MR hypotheses. Finally, most studies on the mechanism of milk’s different effects on bone mineral density in men and women focus on the difference in bone loss. More detailed mechanism studies are still relatively rare, which is also an issue that our team will study in the future.

## Conclusion

According to our study, we found that women who consume more milk have a higher FN-BMD. Women may improve femoral neck bone density and prevent osteoporotic fractures by consuming more milk. When studying the effect of milk consumption on bone density in further studies, we need to pay more attention to women.

## Data Availability Statement

The original contributions presented in the study are included in the article/**Supplementary Material**. Further inquiries can be directed to the corresponding author.

## Author Contributions

SoC, TC participated in Study design. Data was acquired and analyzed by SoC, JC, YP. SYC participated in the study’s supervision. The manuscript was drafted by SoC. CZ has the same contribution to the article as SoC. All authors contributed to the article and approved the submitted version.

## Funding

Science and Technology Plan Project of Fuzhou Science and Technology Bureau in 2019 (No. 2019-SZ-10); Fujian Provincial Clinical Medical Research Center for First Aid and Rehabilitation in Orthopaedic Trauma(2020Y2014); Fuzhou Trauma Medical Center Project (2018080303).

## Conflict of Interest

The authors declare that the research was conducted in the absence of any commercial or financial relationships that could be construed as a potential conflict of interest.

## Publisher’s Note

All claims expressed in this article are solely those of the authors and do not necessarily represent those of their affiliated organizations, or those of the publisher, the editors and the reviewers. Any product that may be evaluated in this article, or claim that may be made by its manufacturer, is not guaranteed or endorsed by the publisher.
