# Supplementary material for: Genetically Predicted Milk Intake Increased Femoral Neck Bone Mineral Density in Women But Not in Men
Source: Front Endocrinol (Lausanne). 2022 Jun 20;13:900109. doi: 10.3389/fendo.2022.900109 (PMC9251187; doi:10.3389/fendo.2022.900109)
Supplement: Supplementary file 1 [file DataSheet_1.docx]

Supplementary Material

**Supplementary Tables**

|  |  |  |  |
| --- | --- | --- | --- |

**Table 1. Sources of data for the primary analysis.**

| **Cohort Full Name** | **Cohort Acronym** | **Ethnicity** | **Femoral Neck BMD** | **Lumbar Spine BMD** |
| --- | --- | --- | --- | --- |
| The Avon Longitudinal Study of Parents and Children | ALSPAC | North‐western European | 3,385 | - |
| TwinsUK | TWINSUK | North‐western European | 4,531 | 4,572 |
| Australasian Osteoporosis Genetics Consortium | AOGC | Caucasian | 2666 | 1923 |
|  |  |  |  |  |
| Framingham Heart Study | FHS | European American | 7096 | 6956 |
|  |  |  |  |  |
| Rotterdam Study‐I | RS‐I | North‐western European | 5340 | 5358 |
|  |  |  |  |  |
| Women's Health Initiative | ESP | Caucasian | 587 | 579 |
| Erasmus Rucphen Family Study | ERF | Caucasian | 1,227 | 1,226 |
| Osteoporotic Fractures in Men USA | MrOS | Non-Hispanic white | 4,581 | 4,550 |
|  |  |  |  |  |
| Australasian Osteoporosis Genetics Consortium | SOF | Non-Hispanic white | 3,322 | 3,334 |
| Barcelona Cohorte Osteoporosis | BARCOS | Mediterranean European | 1,351 | 1,443 |
| Cantabria-Camargo | CABRIO-C | Caucasian (Spanish) | 1,426 | 1,431 |
| Cantabria Osteoporosis Case-control | CABRIO-CC | Caucasian (Spanish) | 1,206 | 1,202 |
| Calcium Intake Fracture Outcome Study | CAIFOS | Caucasian | 1,043 | 1,082 |
| Canadian Multicentre Osteoporosis Study | CAMOS | North-western European | 2,303 | 2,308 |
| Danish Osteoporosis Prevention Study Cohort | DOPS | Northern European | 1,702 | 1,710 |
| Edinburgh Osteoporosis Study | EDOS | British (white caucasian) | 1,762 | 1,966 |
| European Prospective Investigation of Cancer (Norfolk, UK cohort) | EPICNOR | European | 1,372 | - |
| Polish Osteoporosis Study | EPOLOS | European | 664 | 664 |
| Quebec sample | GEOS | Caucasian | 2,376 | 2,377 |
| Malta Osteoporotic Fracture Study | MaltaOFS | Southern European | 1,046 | 1,048 |
| PEAK-25 | Peak25 | Caucasian | 1,002 | 1,002 |
| Total | | | 49,988 | 44,731 |

**Table 2. Sources of data for the sensitivity analysis.**

| **Cohort Full Name** | **Cohort Acronym** | **Ethnicity** | **Femoral Neck**  **BMD** | | **Lumbar Spine BMD** | |
| --- | --- | --- | --- | --- | --- | --- |
|  |  |  | **Men** | **Woman** | **Men** | **Woman** |
| Amish Family Osteoporosis Study | AFOS | Old Order Amish (European Ancestry) | 443 | 475 | 443 | 475 |
| Anglo‐Australasian Osteoporosis Genetics Consortium  high BMD group | AOGC high BMD group | North‐western European | 0 | 1021 | 0 | 618 |
| Anglo‐Australasian Osteoporosis Genetics Consortium  low BMD group | AOGC low BMD group | North‐western European | 0 | 865 | 0 | 552 |
| Cardiovascular Health Study | CHS | European American | 340 | 568 | 340 | 568 |
| DeCODE Genetics Study | DeCODE | North‐western European | 1115 | 6279 | 1135 | 6461 |
|  |  |  |  |  |  |  |
|  |  |  |  |  |  |  |
| Erasmus Rucphen Family | ERF | North‐western European | 1207 | 1512 | 1215 | 1504 |
| Europen Prospective Investigation into Cancer, Norfolk study | EPIC Norfolk | European | 109 | 111 | 0 | 0 |
| Framingham Heart Study | FHS | European American | 1531 | 2043 | 1492 | 2008 |
|  |  |  |  |  |  |  |
| Gothenburg Osteoporosis and Obesity Determinants Study | GOOD | Northern European | 938 | 0 | 938 | 0 |
| Health Aging and Body Composition | HABC | European American | 869 | 776 | 871 | 778 |
| Hong Kong Osteoporosis Study | HKOS | Southern Chinese of Han origin | 0 | 800 | 0 | 800 |
| Indiana Genetics of Bone Fragility Study | Indiana | European American | 0 | 1,479 | 0 | 1479 |
| The Orkney Complex Disease Study | ORCADES | North‐western European | 192 | 229 | 194 | 233 |
| Rotterdam Study‐I | RS‐I | North-western European | 2,106 | 2799 | 2116 | 2798 |
| Rotterdam Study‐II | RS‐II | North‐western European | 779 | 888 | 781 | 898 |
| Rotterdam Study‐III | RS‐III | North‐western European | 511 | 666 | 437 | 583 |
| TwinsUK-1 | TUK‐1 | North‐western European | 0 | 1487 | 0 | 1517 |
| TwinsUK-23 | TUK‐23 | North‐western European | 368 | 2404 | 371 | 2427 |
| Total | | | 10508 | 24402 | 10333 | 23699 |
